# Deciphering the Core Metabolites of Fanconi Anemia by Using a Multi-Omics Composite Network

**DOI:** 10.4014/jmb.2106.06027

**Published:** 2021-12-22

**Authors:** Xiaobin Xie, Xiaowei Chen

**Affiliations:** 1 Department of Pathology, School of Basic Medical Science, Guangzhou Medical University, Guangzhou, Guangdong 511436, P.R. China; 2 Department of Hematology, Guangzhou First People's Hospital, South China University of Technology, Guangzhou 510080, P.R. China

**Keywords:** Prioritized metabolites, Fanconi anemia (FA), multi-omics composite network, MetPriCNet

## Abstract

Deciphering the metabolites of human diseases is an important objective of biomedical research. Here, we aimed to capture the core metabolites of Fanconi anemia (FA) using the bioinformatics method of a multi-omics composite network. Based on the assumption that metabolite levels can directly mirror the physiological state of the human body, we used a multi-omics composite network that integrates six types of interactions in humans (gene-gene, disease phenotype-phenotype, disease-related metabolite-metabolite, gene-phenotype, gene-metabolite, and metabolite-phenotype) to procure the core metabolites of FA. This method is applicable in predicting and prioritizing disease candidate metabolites and is effective in a network without known disease metabolites. In this report, we first singled out the differentially expressed genes upon different groups that were related with FA and then constructed the multi-omics composite network of FA by integrating the aforementioned six networks. Ultimately, we utilized random walk with restart (RWR) to screen the prioritized candidate metabolites of FA, and meanwhile the co-expression gene network of FA was also obtained. As a result, the top 5 metabolites of FA were tenormin (TN), guanosine 5'-triphosphate, guanosine 5'-diphosphate, triphosadenine (DCF) and adenosine 5'-diphosphate, all of which were reported to have a direct or indirect relationship with FA. Furthermore, the top 5 co-expressed genes were CASP3, BCL2, HSPD1, RAF1 and MMP9. By prioritizing the metabolites, the multi-omics composite network may provide us with additional indicators closely linked to FA.

## Introduction

Aplastic anemia (AA) is a bone marrow hematopoietic failure syndrome, characterized mainly by hypoplasia of bone marrow nucleated cells pancytopenia as well as the consequent anemia, hemorrhage and infections [1]. AA is a product of nature or nurture [2]. T lymphocyte abnormal activation, bone marrow injury-triggered hyperthyroidism, hematopoietic cell apoptosis and hematopoietic failure are dominant factors involved in AA pathogenesis [3]. Congenital AA includes Fanconi anemia (FA), congenital dyskeratosis (DKC), Diamond-Blackfan anemia (DBA), and Shwachmann-Diamond syndrome (SDS) [4]. At present, the diagnostic methods of AA rely mainly on blood routine examination, multi-site bone marrow biopsy, bone marrow biopsy, kidney function, thyroid function, blood biochemistry and virological examination, flow cytometry detection, antibodies detection, and imaging examination [5]. However, the diagnostic criteria of AA still rely on blood tests, bone marrow puncture and bone marrow biopsy (iliac), all of which are detrimental to patients. In order to find an efficient and noninvasive diagnostic method, further investigations are required to elucidate the underlying mechanisms of AA.

Metabolomics has emerged as an essential technology platform used to comprehend the underlying mechanisms of diseases and to discover relevant biomarkers through detecting composition and changes of endogenous small molecule metabolites [6]. By dint of the intrinsic sensitivity of metabolomics, we can detect the subtle changes in biological pathways which could provide insight into the mechanisms underlying the disease [7]. In addition, various analytical methods, for example liquid chromatography-mass spectrometry (LC-MS), gas chromatography-mass spectrometry (GC-MS) and nuclear magnetic resonance (NMR) used in metabolomics can be used to analyze biological samples continuously and nondestructively at any time [8].

Systemic biology takes a holistic "omics study" analysis of genes, proteins and metabolites to explore the effects of assorted exogenous stimuli such as drugs, diseases, gene modifications, nutrition and environment factors on the biological system. To this end, we utilized a method named “MetPriCNet” proposed by Yao *et al*. [9] to forecast and prioritize candidate metabolites of FA based on the multi-omics composite network, which has a high area under the curve (AUC) value than other methods even without metabolite information. MetPriCNet prioritizes candidate metabolites in line with their multi-omics composite networks with seed nodes, which integrate information from the phenome, genome, and metabolome as well as their interactions. Finally, we sorted out the preferred metabolites of FA using MetPriCNet and obtained the relationship between preferred metabolites and co-expressed genes of FA.

## Material and Methods

**Fig. 1:**
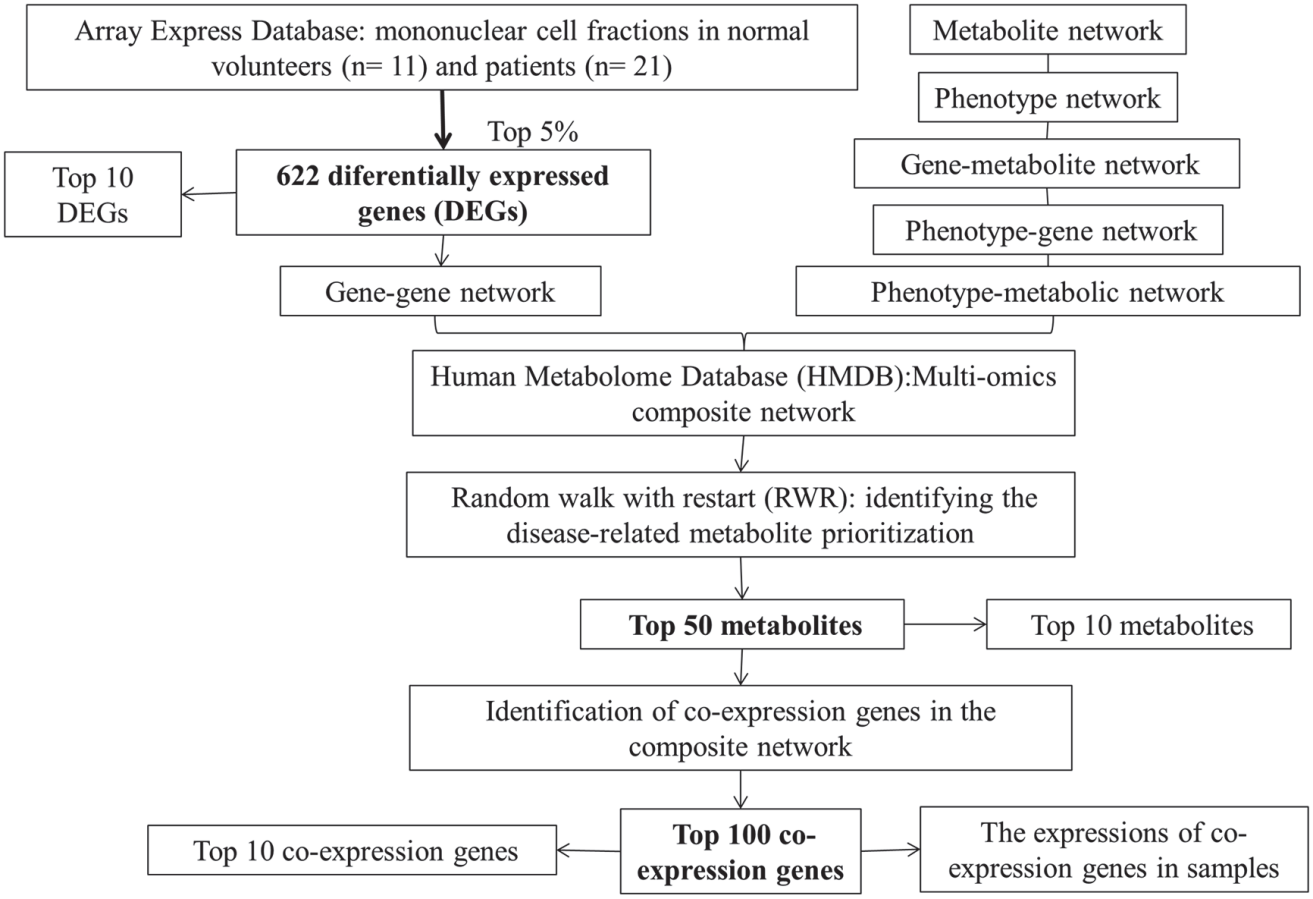
An algorithm flowchart.

The detailed information for each step in the present analysis was described in an algorithm flowchart (Fig. 1).

### Gene Expression Data and Disease Information

The expression data of 11 healthy volunteers and 21 FA patients with low-density mononuclear cell fractions were obtained from the ArrayExpress database (https://www.ebi.ac.uk/arrayexpress/) with the accession number E-GEOD-16334, which excluded all FA patients with clonal evolutionary cytogenetic evidence and acute leukemia.

Post pretreatment of these data, the expression profile data of 12,437 genes were acquired by mapping between the probe and the gene. By obtaining access to Online Mendelian Inheritance in Man (OMIM) via 609135, ID of FA , we acquired the data collected from this platform and obtained the seed genes related to FA, including ABCB7, ALAS2, BRCA2, BRIP1, CAD, CSF2, ERCC4, FANCA, FANCB, FANCC and FANCD2 RPL11, RPL15, RPL26, RPL35A, RPL5, RPS10, RPS17, RPS19, RPS24, RPS26, RPS15, RPS29, RPS7, SBDS, SLC25A38, SLX4, SOD2, TERT, TNF and UMPS. However, the FA-related seed metabolites are unknown. The samples were divided into control group and experimental group. The different expressions of genes were calculated by limma package. *T*-test and *F*-test were performed on the gene expression matrix. The lmFit function was used to linearly fit the data. eBayes statistics and false discovery rate (FDR) were applied to adjust the *p*-value. The genes were sorted by *p*-value, and the top 5% of the genes were selected as differential genes and used to build a genetic network.

### Comprehensive Multi-Omics Information

The known disease metabolites were obtained from the Human Metabolome Database (HMDB, http://www.hmdb.ca/). The known disease gene information was extracted from the Morbid map file of the OMIM database (https://omim.org/), which contained a comprehensive description of the human genes and phenotypes as well as their associations. A multi-omics composite network was constructed through the integration of six datasets, which consisted of six networks termed i) genetic network, ii) metabolic network, iii) phenotypic network, iv) gene-metabolite action network, v) phenotype-gene action network and vi) phenotype-metabolic interaction network.

### Multi-Omics Information of AA

The multi-omics information on AA was acquired through enriching differential genes to the obtained comprehensive multi-omics information.

### Construction of the Multi-Omics Composite Network

In order to establish a multi-omics composite network, the above-mentioned six types of network data were integrated into composite network A.

$$A=\left[\begin{array}{ccc} A_{G} & A_{GP} & A_{GM}\\ A_{GP}^{T} & A_{P} & A_{PM}\\ A_{GM}^{T} & A_{PM}^{T} & A_{M} \end{array}\right]$$

W represents the transition matrix of composite network A, which can be deduced from the adjacency with matrix A.

$$W=\left[\begin{array}{ccc} W_{G} & W_{GP} & W_{GM}\\ W_{PG} & W_{P} & W_{PM}\\ W_{MG} & W_{MP} & W_{M} \end{array}\right]$$

W_ij_ signifies the probability of transition from node i to node j. x, y and z are the probability of transition between the gene network and the phenotype network, the gene network and the metabolic network, as well as the phenotypic network and the metabolic network, respectively. The default value is 1/3. The probability of gene i (g_i_) to gene j (g_j_) in the genetic network can be defined as follows:

$$W_{G}(i,j)=P(g_{j}ㅣg_{i})=\{\begin{array}{cc} (1-x-y)A_{G}(i,j)/\sum {}_{j}A_{G}(i,j), & if\sum {}_{j}A_{GP}(i,j)\neq 0\text{ and }\sum {}_{j}A_{GM}(i,j)\neq 0\;\\ (1-x)A_{G}(i,j)/\sum {}_{j}A_{G}(i,j), & if\sum {}_{j}A_{GP}(i,j)\neq 0\text{ and }\sum {}_{j}A_{GM}(i,j)=0\;\\ (1-y)A_{G}(i,j)/\sum {}_{j}A_{G}(i,j), & if\sum {}_{j}A_{GP}(i,j)=0\text{ and }\sum {}_{j}A_{GM}(i,j)\neq 0\;\\ A_{G}(i,j)/\sum {}_{j}A_{G}(i,j), & if\sum {}_{j}A_{GP}(i,j)=0\text{ and }\sum {}_{j}A_{GM}(i,j)=0\; \end{array}.$$

Similarly, the transition probability from gene i (g_i_) to phenotype j (p_j_) is defined as follows:

$$W_{GP}(i,j)=\text{P}(p_{j}ㅣg_{i})=\{\begin{array}{cc} xA_{GP}(i,j)/\sum {}_{j}A_{GP}(i,j), & if\sum {}_{j}A_{GP}(i,j)\neq 0\\ 0, & otherwise \end{array}.$$

The transition probability from gene i (g_i_) to metabolite j (m_j_) is defined as follows:

$$W_{GM}(i,j)=\text{P}(m_{j}ㅣg_{i})=\{\begin{array}{cc} yA_{GM}(i,j)/\sum {}_{j}A_{GM}(i,j), & if\sum {}_{j}A_{GM}(i,j)\neq 0\\ 0, & otherwise \end{array}.$$

In the phenotypic network, the probability from the phenotype i (p_i_) to the phenotype j (p_j_) is defined as follows:

$$W_{P}(i,j)=P(p_{j}ㅣp_{i})=\{\begin{array}{cc} (1-x-z)A_{P}(i,j)/\sum {}_{j}A_{P}(i,j), & if\sum {}_{j}A_{PM}(i,j)\neq 0\;and\;\sum {}_{j}A_{GP}(j,i)\neq 0\;\\ (1-z)A_{P}(i,j)/\sum {}_{j}A_{P}(i,j), & if\sum {}_{j}A_{PM}(i,j)\neq 0\;and\;\sum {}_{j}A_{GP}(j,i)=0\;\\ (1-x)A_{P}(i,j)/\sum {}_{j}A_{P}(i,j), & if\sum {}_{j}A_{PM}(i,j)=0\;and\;\sum {}_{j}A_{GP}(j,i)\neq 0\;\\ A_{P}(i,j)/\sum {}_{j}A_{P}(i,j), & if\sum {}_{j}A_{PM}(i,j)=0\;and\;\sum {}_{j}A_{GP}(j,i)=0\; \end{array}.$$

The probability from phenotype i (pi) to gene j (gj) is defined as follows:

$$W_{PG}(i,j)=P(g_{j}ㅣp_{i})=\{\begin{array}{cc} xA_{GP}(j,i)/\sum {}_{j}A_{GP}(j,i), & if\sum {}_{j}A_{GP}(j,i)\neq 0\\ 0, & otherwise \end{array}.$$

The probability from phenotype i (pi) to metabolite j (mj) is defined as follows:

$$W_{PM}(i,j)=P(m_{j}ㅣp_{i})=\{\begin{array}{cc} zA_{PM}(i,j)/\sum {}_{j}A_{PM}(i,j), & if\sum {}_{j}A_{PM}(i,j)\neq 0\\ 0, & otherwise \end{array}.$$

In the metabolite network, the probability from metabolite i (mi) to metabolite j (m) is defined as follows:

$$W_{M}(i,j)=P(m_{j}ㅣm_{i})=\{\begin{array}{cc} (1-y-z)A_{M}(i,j)/\sum {}_{j}A_{M}(i,j), & if\sum {}_{j}A_{GM}(j,i)\neq 0\;and\;\sum {}_{j}A_{PM}(j,i)\neq 0\;\\ (1-y)A_{M}(i,j)/\sum {}_{j}A_{M}(i,j), & if\sum {}_{j}A_{GM}(j,i)\neq 0\;and\;\sum {}_{j}A_{PM}(j,i)=0\;\\ (1-z)A_{M}(i,j)/\sum {}_{j}A_{M}(i,j), & if\sum {}_{j}A_{GM}(j,i)=0\;and\;\sum {}_{j}A_{PM}(j,i)\neq 0\;\\ A_{M}(i,j)/\sum {}_{j}A_{M}(i,j), & if\sum {}_{j}A_{GM}(j,i)=0\;and\;\sum {}_{j}A_{PM}(j,i)=0\; \end{array}.$$

The probability from metabolite i (mi) to gene j (gj) is defined as follows:

$$W_{MG}(i,j)=P(g_{j}ㅣm_{i})=\{\begin{array}{cc} yA_{GM}(j,i)/\sum {}_{j}A_{GM}(j,i), & if\sum {}_{j}A_{GM}(j,i)\neq 0\\ 0, & otherwise \end{array}.$$

The probability from metabolite i (mi) to phenotype j (pj) is defined as follows:

$$W_{MP}(i,j)=P(p_{j}ㅣm_{i})=\{\begin{array}{cc} zA_{PM}(j,i)/\sum {}_{j}A_{PM}(j,i), & if\sum {}_{j}A_{PM}(j,i)\neq 0\\ 0, & otherwise \end{array}.$$

### Random Walk with Restart (RWR)

To obtain the preferred candidate metabolites in the complex network, we utilized the RWR method to extend the screening to a multithreaded composite network. The method selected the preferred candidate metabolite as per the proximity of every candidate to the seed candidate metabolite (i.e., the known metabolite) and simulated a random walk from the seed node. Every step of the walk moved from the current node to its immediate neighbor at probability 1-α, or returned to the seed node at probabilityα. The formula for the calculation is as follows:

$$P^{k+1}=(1-\alpha )WP^{k}+\alpha P^{0}.$$

Here, P^0^ represents the beginning probability vector. P^k^ manifests the probability vector at which the i-th element is held at node I and the alpha default value is 0.7. W signifies the transition matrix of the composite network A.

(1) initial probability vector P^0^

u^0^, v^0^ and w^0^ were supposed to be the beginning probabilities of the genetic network, the phenotype network and the metabolic network, respectively. For a phenotype (i.e. disease), the seed nodes comprised phenotypes, corresponding known metabolites and known genes. The phenotypes that were associated with FA included FA of complementation group V (FANCV, caused by mutation in the REV7 gene on chromosome 1p36), FA of complementation group T (FANCT, caused by compound heterozygous mutation in the UBE2T gene on chromosome 1q32), FA of complementation group L (FANCL, caused by homozygous or compound heterozygous mutation in the PHF9 gene on chromosome 2p16), FA of complementation group D2 (FANCD2, caused by compound heterozygous or homozygous mutation in the FANCD2 on chromosome 3p25), etc. The initial probability of the genetic network u0 was computed by giving an equal probability to the gene nodes in the gene network. The sum was equal to 1, meaning that the random walk began at the same probability from each seed node. Similarly, the initial probabilities v0 and w0 were calculated, where a = 1/3, and b = 1/3.

$$P^{0}=\left|\begin{array}{c} a*v_{0}\\ b*v_{0}\\ (1-a-b)*w_{0} \end{array}\right|$$

(2) k-step probability vector P^k^

After multiple iterations, the change between P^k + 1^ and P^k^ was less than 10^-10^, where the probability reached a steady state and the iteration stopped.

### Identifying Preferred Metabolites and Their Co-Expressed Genes

After the random walk reached the steady state, each metabolite in the composite network had a corresponding probability. After removal of the seed metabolite nodes, the metabolites were sorted according to the probability. The top 50 metabolites were screened as the preferred metabolites. The disease-preferred metabolite information was derived from the multi-group composite network, and the disease-preferred metabolite network was screened out. We identified the genes that interacted with the preferred metabolites, and then analyzed and selected the genes having a score greater than the mean and ranking in the top 100 as co-expressed genes.

## Results

### Candidate Differential Genes of FA

By gauging the *p*-values, the top 5% of the genes were selected, and a total of 622 candidate differential genes were finally obtained to implement the gene-gene network. The top 10 differential genes were KIT, CPA3, PPM1H, IRAK3, RNASEH2A, CDK4, NREP, ARMCX1, MYB and RAB13 (Table 1).

### Multi-Omics Composite Network of Human

The concrete data of the human multi-omics composite network comprising six networks were delineated in Table 2. We constructed an AA-related composite multi-omics network containing the interactions of gene-gene, phenotype-phenotype, metabolite-metabolite, gene-phenotype, gene-metabolite, and phenotype-metabolite by importing the gene network.

Gene network (A_G_): we downloaded the human protein interaction network data from STRING (containing 10,48576 interactions), converted the protein ID to the gene name, and removed the repetitive interaction pair to obtain a protein-protein interaction (PPI) gene network involving 16,785 nodes and 1,515,370 pairs of interactions.

Metabolite network (A_M_): first, metabolic pathways were collected from KEGG and HMDB, and 4,994 human metabolites were harvested from human metabolite pathways obtained from Reactome, MSEA and SMPDB. We then analyzed the interrelationships between human metabolites from STITCH, which must be included in 4,994 human metabolites. Eventually, 3,764 human metabolites and 74,667 interactions between human metabolites were acquired (not all metabolites were associated with STITCH).

Phenotype network (A_P_): the phenotypic network was established according to phenotype-phenotype relationships reported by Van Driel *et al*. [10].

Gene-metabolite association network (A_GM_): we used the STITCH database to obtain chemical/metabolite-gene associations. Based on 4,994 kinds of human metabolites, the metabolites not existing in the metabolic network and genes not included in gene networks were filtered. At last, a total of 12,342 genes, 3,278 kinds of metabolites and 192,763 pairs of gene-metabolite interactions were obtained.

Phenotype-gene association network (A_GP_): we obtained the phenotype-gene relationship from OMIM's planned morbid map file. The phenotype that was not in our phenotypic network and the genes which were not in our gene network were filtered. A total of 2,603 interactions between 1,886 phenotypes and 1,715 genes were acquired.

Phenotype-metabolite association network (A_PM_): we collected the phenotype-metabolite association from HMDB. Following filtration, 664 pairs of interactions between 149 phenotypes and 388 metabolites were retained. Consequently, we procured 25,629 nodes and 11,926,113 edges involved in AA-related DEGs, phenotypes and metabolites.

### Identifying the FA-Related Metabolite Prioritization

**Fig. 2:**
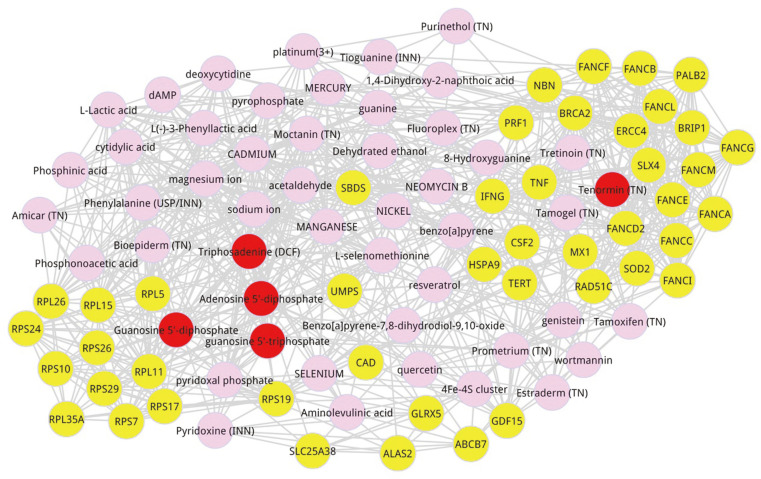
Disease-preferred metabolite composite network. Prioritization of AA-related metabolite network selected by multi-omics composite network. The pink nodes represent the metabolites, the red nodes signify the top 5 metabolites, and the yellow indicates the seed nodes.

The multi-omics information of FA was obtained from enriched differential genes to multi-omics composite network of human, and the result was described in Table 3. The prioritization of the FA-related metabolites can be identified by evaluating the relation score of each metabolite in the multi-omics composite network. By combining the original weight score that was calculated with the RWR method, the new relation score of each metabolite was computed, and the metabolite was further ranked by the corresponding score. In the present study, we selected 50 metabolites as prioritized candidate metabolites which ranked in the top 50 in terms of relation scores. The top 10 metabolites were displayed in Table 4. The disease-preferred metabolite information was collected from the multi-group composite network, and the disease-preferred metabolite network was screened out (Fig. 2), where the top 5 metabolites with a higher score were marked in red, including tenormin (TN) (score = 0.000677293), guanosine 5'-triphosphate (score = 0.002984), guanosine 5'-diphosphate (score = 0.002867), and triphosadenine (DCF) (score = 0.002808), adenosine 5'-diphosphate (score = 0.002593). Seed nodes were marked in yellow and other metabolites were in pink.

### Identification of Co-Expressed Genes in the Composite Network

Clustergrammer was applied to visualize the co-expressed gene expression data.

**Fig. 3:**
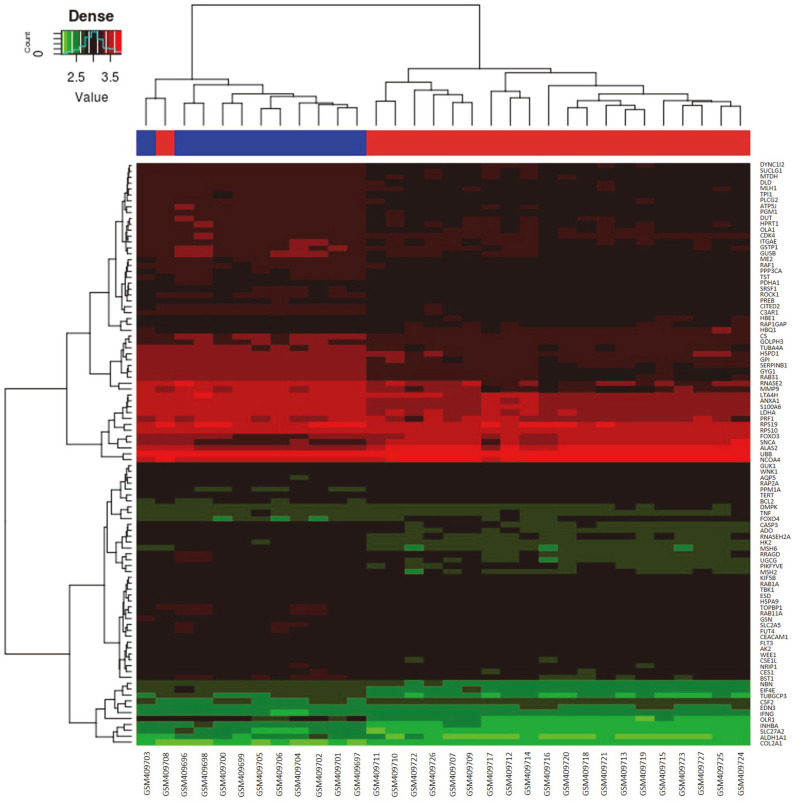
Heatmap of AA-preferred metabolite co-expressed gene. A heatmap of the gene expression was generated using the R-package pheatmap. Gene expression profiles were displayed with 100 co-expressed genes in rows and samples in columns. Green to red represented the spectrum from low to high. Blue represented normal group. Red represented patients group.

According to the 50 prioritized candidate metabolites, genes interacting with metabolites in the composite network were chosen. Moreover, we identified genes that interacted with 50 prioritized candidate metabolites and analyzed their score values to single out the genes having a score value greater than the mean and ranking in top 100 as co-expressed genes. Further, two groups of samples with 100 preferred metabolite co-expressed gene expressions (rows) were showed in the a heatmap of FA (Fig. 3). Of those, 22 genes were upregulated, and 77 others were downregulated in patients group. The SLX4 gene was not found in the dataset, which was not present in Fig. 3.

**Fig. 4:**
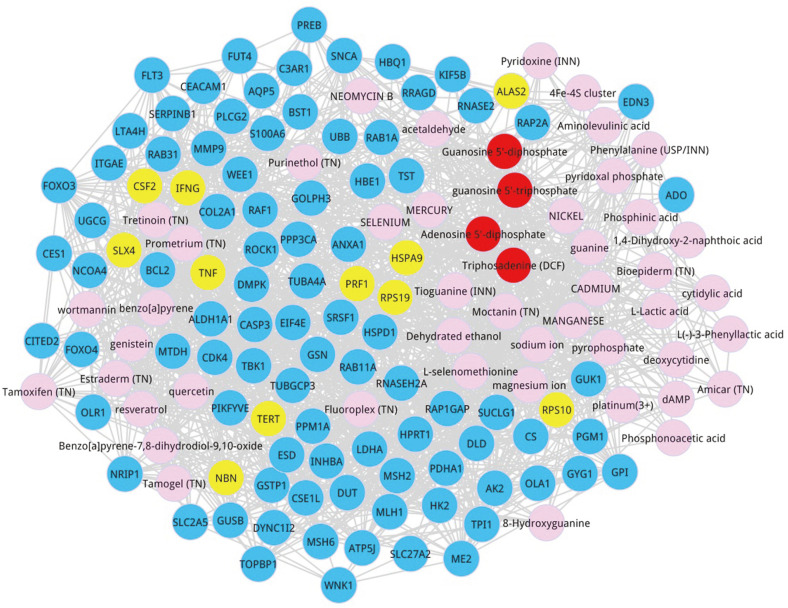
Disease-preferred metabolite co-expressed gene distribution. Co-expressed network related to the top 50 metabolites, seed nodes and co-expressed genes. The blue node represented the co-expression gene, the pink node represented the prioritized candidate metabolite, the yellow represented the seed node and red nodes were the top 4 metabolites.

As a result, a co-expressed network was obtained (Fig. 4). In this composite network, yellow nodes represented the seed nodes, blue nodes manifested the co-expressed genes, pink nodes signified the prioritized candidate metabolites and red nodes indicated the top 4 metabolites. The parameter information on the 10 co-expressed genes with a degree greater than 30 was listed in Table 5, including CASP3, BCL2, TNF, HSPD1, RAF1, MMP9, IFNG, HPRT1, LDHA, and DUT.

## Discussion

With the assistance of a combined multiomics analysis, we screened out the preferred metabolites of FA, and analyzed the relationship between preferred metabolites and co-expressed genes of FA. The top 5 metabolites of FA were TN, guanosine 5'-triphosphate, guanosine 5'-diphosphate, DCF, and adenosine 5'-diphosphate. The top 5 co-expressed genes were CASP3, BCL2, HSPD1, RAF1 and MMP9. The successful sorting of preferred metabolites can be attributed to a model of a multi-omics composite network, which depends on several aspects. Initially, we utilized a composite network comprising six networks, that is, genome, phenome, metabolome, gene-metabolite action network, phenotype-gene action network, and phenotype-metabolite interaction network. Secondly, this multi-omics composite network exploited the advantage of RWR method to capture the global multi-omics information. It maintained that the candidate metabolites were ordered according to the interaction information in the whole composite network, but not only the local environment.

The biological system can be reflected in some aspects, such as genome, metabolome, phenome, and interactome information which integrate into a composite network. Therefore, constructing a network based on multi-omics information might be useful in finding disease-related risk metabolites. A multi-omics composite network, named MetPriCNet, was first used for predicting and prioritizing candidate metabolites by Yao *et al*.[11]. The novel integrated network was established on the basis of six data sources. From a perspective of integrating multi-omics information, MetPriCNet has an advantage over RWR only in the metabolite network (PROFANCY) [12]. MetPriCNet could achieve a higher AUC value than PROFANCY, which indicated that MetPriCNet upgraded the performance by integrating the multi-omics information. Furthermore, MetPriCNet could prioritize the candidate metabolites, even in the absence of disease metabolites, using other known information such as related disease genes and phenotypes [13]. The robustness of MetPriCNet was assessed by introducing noise into the network weight score. As a result, MetPriCNet achieved an AUC value above 0.79, although the relation score was disturbed by up to 30% noise, which implied that this multi-omics composite network was more robust to noise than a single PPI network [9]. Thus, in this report, we utilized the MetPriCNet method to select preferred metabolites and their co-expressed genes.

TN is a beta blocker that is used to cure angina (chest pain) and hypertension (high blood pressure), and is also prescribed to lower the risk of death after a heart attack [14]. This result reminds us that patients with angina or hypertension have a higher possibility of suffering from FA. Guanosine could reverse anemia condition induced by mycophenolic acid, which hints that guanosine may be an inhibitor of AA [9]. Recently, the altered morphology of the mitochondrion, the principal site of aerobic ATP (adenosine triphosphate) production, and its deficiency of energetic activity in FA cells was reported [9]. DCF and adenosine 5'-diphosphate are different forms of ATP. Accordingly, increased energy supply from red blood cells in the form of ATP could improve anemia-induced hemolysis [9]. In this report, although the selected top 5 metabolites have been rarely reported on in relation to FA, their relationships with anemia have been mostly investigated. Some of the top 5 co-expressed genes were also directly or indirectly related with AA. *Dioscorea nipponica* Makino could alleviate AA by suppressing the expression of intracellular apoptosis protein, caspase-3 [15]. Upregulation of Bcl-2 protein denotes chemoresistance in acute myeloid leukemia. HSPD1 and RAF1 have not been reported to be related with AA or relevant diseases. Salidroside could facilitate the hematopoietic function recovery of bone marrow depressed anemic mice by promoting the expression and activity of MMPs [13]. MMP inhibitors were used in the management of FA immortalized fibroblasts [16]. However, despite these contributions, the present study has limitation. As Fanconi anemia is a congenital form of anemia, it would be significantly associated with genetic variants, but an ideal data set was lacking in this study. Despite this lack of data, this study has important implications.

The multi-omics composite network in screening the metabolites was verified to be able to provide us with additional indicators closely linked to AA. These indicators of FA were not usually to be found. However, in this report, by introducing multi-omics composite network methods, and utilizing the perfect combination of the computing power of computer and statistics, we could comprehensively find AA-related metabolites, and further screen out the most closely linked top 5 metabolites. This provides a more reliable basis for future diagnosis of FA and the possibility of noninvasive diagnosis of FA. However, biological experiments are urgently needed to facilitate the conversion of our results into clinical application.

## Figures and Tables

**Table 1:** Differential candidate gene results.

	logFC	AveExpr	t	*p* Value	adj.P.Val	B
KIT	-0.25824	2.762042	-12.7503	1.57E-14	1.23E-10	23.03051
CPA3	-0.27275	2.999178	-12.6477	1.98E-14	1.23E-10	22.80946
PPM1H	-0.15461	2.198137	-12.0878	7.04E-14	2.44E-10	21.57958
IRAK3	-0.3328	2.534542	-11.9707	9.22E-14	2.44E-10	21.31721
RNASEH2A	-0.12468	2.795899	-11.9444	9.80E-14	2.44E-10	21.25805
CDK4	-0.12	3.187248	-11.6921	1.77E-13	3.44E-10	20.68571
NREP	-0.21505	2.994259	-11.5552	2.44E-13	3.44E-10	20.3717
ARMCX1	-0.17256	2.113454	-11.4925	2.83E-13	3.44E-10	20.22703
MYB	-0.26273	2.711663	-11.4822	2.90E-13	3.44E-10	20.20342
RAB13	-0.25063	3.047814	-11.4775	2.93E-13	3.44E-10	20.1925

**Table 2:** The statistics of the human composite network.

Statistics of the composite network	Node	Edge
Gene network	16785	1515370
Metabolite network	3764	74667
Phenotype network	5080	10140046
Gene-metabolite association network	12342 genes, 3278 metabolites	192763
Phenotype-gene association network	1886 phenotypes, 1715 genes	2603
Phenotype-metabolite association network	149 phenotypes, 388 metabolites	664
All	25629	11926113

**Table 3:** The statistics of AA multi-omics network.

Statistics of the composite network	Node	Edge
Gene network	621	9490
Metabolite network	3764	74667
Phenotype network	5080	10140046
Gene-metabolite association network	621 genes, 3764 metabolites	11756
Phenotype-gene association network	5080 phenotypes, 621 genes	136
Phenotype-metabolite association network	149 phenotypes, 388 metabolites	664
Total	9465	10236759

**Table 4:** The preferred top 10 metabolites of AA.

Rank	Metabolite CID	Metabolite name	Score
1	2249	Tenormin (TN)	0.004441
2	6830	Guanosine 5'-triphosphate	0.002984
3	8977	Guanosine 5'-diphosphate	0.002867
4	5957	Triphosadenine (DCF)	0.002808
5	6022	Adenosine 5'-diphosphate	0.002593
6	753	Moctanin (TN)	0.002097
7	6398953	4Fe-4S cluster	0.001386
8	23931	MERCURY	0.001227
9	5280961	Genistein	0.000911
10	5757	Estraderm (TN)	0.000908

**Table 5:** Preferred top10 co-expression genes of metabolites of AA.

Rank	Gene Name	Degree	Closeness	Betweenness	Transitivity
1	CASP3	65	1.29492631	11	0.264904
2	BCL2	58	1.272537967	10	0.272837266
3	TNF	54	1.439664902	91	0.209643606
4	HSPD1	49	1.373017665	70	0.306122449
5	RAF1	43	1.281777937	8	0.321151717
6	MMP9	43	1.178360996	0	0.318936877
7	IFNG	43	1.330200416	108	0.297895903
8	HPRT1	43	1.479520582	294	0.246954596
9	LDHA	39	1.350209521	19	0.313090418
10	DUT	39	1.244110835	87	0.330634278

The degree is the number of edges connected to each node; closeness represents the closeness between a node and other nodes in the network; betweenness is a centrality measure of a node within a network; transitivity is a notion measuring the probability that the adjacent nodes of a node are connected among them.

## References

[1] Baltierra D, Harper T, Jones MP, Nau KC (2015). Hematologic disorders: Bone marrow failure. FP Essent..

[2] Yang N, Chen J, Zhang H, Dai Z, Yao H, Ma X (2017). Horse versus rabbit antithymocyte globulin in immunosuppressive therapy of treatment-naive aplastic anemia: a systematic review and meta-analysis. Annal. Hematol..

[3] Liu C, Zheng M, Zhang T, Fu R, Wang H, Wang T (2017). TRAIL in CD8+ T cells from patients with severe aplastic anemia. Int. J. Hematol..

[4] Steele JM, Sung L, Klaassen R, Fernandez CV, Yanofsky R, Wu J (2006). Disease progression in recently diagnosed patients with inherited marrow failure syndromes: a Canadian Inherited Marrow Failure Registry (CIMFR) report. Pediatr. Blood Cancer.

[5] Juárez-Rendón KJ, Rivera Sánchez G, Reyes-López M, García-Ortiz JE, Bocanegra-García V, Guardiola-Avila I (2017). Alopecia areata. current situation and perspectives. Arch. Argent. Pediatr..

[6] Reppe S, Datta HK, Gautvik KM (2017). Omics analysis of human bone to identify genes and molecular networks regulating skeletal remodeling in health and disease. Bone.

[7] Johnson CH, Ivanisevic J, Siuzdak G (2016). Metabolomics: beyond biomarkers and towards mechanisms. Nat. Rev. Mol. Cell Biol..

[8] Stewart JL, Besenyi GB, Williams LB, Burt V, Anglin JC, Ghamande SA (2017). Healthy lifestyle intervention for African American uterine cancer survivors: Study protocol. Contemp. Clin. Trials Commun..

[9] Yao Q, Xu Y, Yang H, Shang D, Zhang C, Zhang Y (2015). Global prioritization of disease candidate metabolites based on a multiomics composite network. Sci. Rep..

[10] Driel MAV, Bruggeman J, Vriend G, Han GB, Leunissen JAM (2006). A text-mining analysis of the human phenome. Eur. J. Hum. Genet..

[11] Simón-Manso Y, Lowenthal MS, Kilpatrick LE, Sampson ML, Telu KH, Rudnick PA (2013). Metabolite profiling of a NIST Standard Reference Material for human plasma (SRM 1950): GC-MS, LC-MS, NMR, and clinical laboratory analyses, libraries, and web-based resources. Anal. Chem..

[12] Moreno-Sánchez R, Saavedra E, Gallardo-Pérez JC, Rumjanek FD, Rodríguez-Enríquez S (2016). Understanding the cancer cell phenotype beyond the limitations of current omics analyses. FEBS J..

[13] Unni S, Yao Y, Milne N, Gunning K, Curtis JR, Lafleur J (2015). An evaluation of clinical risk factors for estimating fracture risk in postmenopausal osteoporosis using an electronic medical record database. Osteoporos. Int..

[14] Hocht C, F MB, J SDM, Santander Plantamura Y, C AT, A HP (2017). What is the real efficacy of beta-blockers for the treatment of essential hypertension?. Curr. Pharm. Design.

[15] Shang D, Li C, Yao Q, Yang H, Xu Y, Han J (2014). Prioritizing candidate disease metabolites based on global functional relationships between metabolites in the context of metabolic pathways. PLoS One.

[16] Rual JF, Venkatesan K, Hao T, Hirozane-Kishikawa T, Dricot A, Li N (2005). Towards a proteome-scale map of the human protein-protein interaction network. Nature.

